# Positive Psychology, Activation, and Self-Care Adherence in a Diverse Sample of Adults With Heart Failure

**DOI:** 10.1093/geroni/igab046.210

**Published:** 2021-12-17

**Authors:** Rebecca Meraz, Jocelyn McGee, Elizabeth Perry Caldwell, Kathryn Osteen

**Affiliations:** 1 Baylor University, Dallas, Texas, United States; 3 Baylor University Louise Herrington School of Nursing, Dallas, Texas, United States; 4 Baylor University Louise Herrington School of Nursing, Baylor University Louise Herrington School of Nursing, Texas, United States

## Abstract

Heart failure (HF) self-care is vital to health and wellbeing, yet more than half of all persons with HF do not adhere to the self-care recommendations of taking medications as prescribed, weighing daily, eating low salt foods, or exercising. It has been suggested that disparities in HF among racial/ethnic groups may be reflective of underlying determinants of health, such as poor engagement in self-care activities, rather than genetic or physiological differences. The purpose of this study was to examine direct and indirect effects of perceived social support, positive psychological (PP) characteristics, and patient activation on self-care behaviors in a diverse sample of older adults with HF. A nationwide survey was conducted in cooperation with the recruitment and sampling company Qualtrics. Stratified random sampling was used where 49% of the 174 respondents were persons of color (POC). The mean age was 60. Logistic regression statistical models were used with a lasso procedure. In this study, PP characteristics and activation level were most predictive of HF self-care adherence, particularly medication adherence. Respondents who were resilient, hopeful, and activated also reported higher medication and self-care adherence. Perceived social support and health literacy levels were not associated with self-care adherence. There were no differences in predictive variables by race/ethnicity, gender, or age. Interventions aimed at increasing resilience, hope, and engagement in care or activation may improve HF self-care adherence among persons with HF. Further research is needed to understand the impact of PP characteristics and patient activation level on HF self-care adherence in POC.

